# Efficacy of Pudendal Nerve Blocks and Ultrasound-Guided Superior Hypogastric Plexus Blocks for the Management of Refractory Interstitial Cystitis: A Case Series

**DOI:** 10.7759/cureus.37709

**Published:** 2023-04-17

**Authors:** Arun Kalava, Matthew Crowley, Gina Parsonis, Lucas Wiegand

**Affiliations:** 1 Anesthesiology, University of Central Florida College of Medicine, Orlando, USA; 2 Physical Therapy, Foundation Physical Therapy, Clearwater, USA; 3 Department of Urology, University of South Florida, Tampa, USA

**Keywords:** nocturia, urinary urgency, urinary frequency, chronic pelvic pain, ultrasound guided nerve block, superior hypogastric plexus, pudendal nerve, bladder pain syndrome, interstitial cystitis

## Abstract

Interstitial cystitis/bladder pain syndrome (IC/BPS) is characterized by chronic pelvic, perineal, or bladder pain in addition to lower urinary tract symptoms. The etiology of this condition is not fully understood, which presents a challenge for effective therapeutic intervention. Current treatment guidelines recommend the use of multimodal pain management strategies including behavioral/non-pharmacologic, oral medications, bladder instillations, procedures, and major surgery. However, the safety and efficacy of these modalities vary, and there is currently no optimal treatment for the management of IC/BPS. The pudendal nerves and superior hypogastric plexus, which together mediate visceral pelvic pain and bladder control, are not addressed in the current guidelines but may serve as a therapeutic target. Here, we report improvements in pain, urinary symptoms, and functionality following bilateral pudendal nerve blocks and/or ultrasound-guided superior hypogastric plexus blocks in three patients with refractory IC/BPS. Our findings provide support for the use of these interventions in patients with IC/BPS unresponsive to prior conservative management.

## Introduction

Interstitial cystitis/bladder pain syndrome (IC/BPS) is a chronic condition characterized by pelvic, perineal, or bladder pain with symptoms of urinary urgency, frequency, or nocturia, in the absence of infectious etiology and abnormal cytology [1]. It comprises a major component of patients with chronic pelvic pain, in addition to other conditions such as endometriosis in women and prostatitis in men [2,3]. Diagnosis is typically made by exclusion of other urinary tract diseases, though the underlying pathophysiology remains incompletely understood.

Despite multiple modalities available for the management of IC/BPS, including behavioral, non-pharmacologic, pharmacologic, and interventional procedures, no optimal therapy currently exists. The American Urological Association (AUA) has recently transitioned its guidelines from a tier-based stepwise approach to the use of multimodal pain management options [4]. The modest levels of efficacy of the aforementioned management options, in addition to the increased risk of poor postoperative outcomes with more invasive procedures, leave much to be desired for patients with IC/BPS.

Peripheral nerve blocks of the pudendal nerves or superior hypogastric plexus, which together mediate visceral pelvic pain and bladder control, may be an alternative therapeutic option. There are reports detailing the efficacy of pudendal nerve blocks and superior hypogastric plexus blocks for the management of chronic pelvic pain in the setting of cancer, endometriosis, and other conditions [5-7]. However, limited evidence exists for the use of these therapeutic modalities in the management of IC/BPS. Here, we report improvements in pain, urinary symptoms, and functionality following pudendal nerve blocks and/or superior hypogastric plexus blocks in three patients with IC/BPS refractory to conservative therapy.

## Case presentation

Procedural techniques

Ischiorectal Approach to Bilateral Pudendal Nerve Block

**Figure 1:**
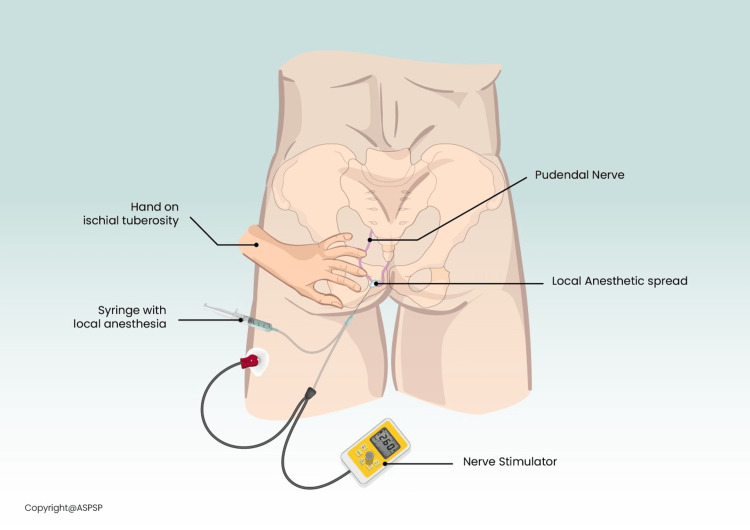
Anatomical illustration of the ischiorectal approach to pudendal nerve block Permission for use granted by the American Society for Post Surgical Pain

The patient was brought to the procedure room, placed in a prone position, and sedated with intravenous propofol. The area was prepped with 2% chlorhexidine gluconate and 70% isopropyl alcohol and draped in a sterile fashion. Injection of 3 ml of 1% lidocaine was used to anesthetize the skin. Using a 22-gauge 3 ½ inch stimulating needle, the left pudendal nerve was first targeted via the ischiorectal approach (Figure 1).

Once there was a visible anal twitch at 1.5 mA and negative aspiration of blood, 5 ml of 0.5% ropivacaine plus 10 mg of dexamethasone was injected (Video 1). The same procedure was repeated on the right side.

**Video 1:** Demonstration of the anorectal twitch for pudendal nerve block Permission for use granted by the American Society for Post Surgical Pain

Ultrasound-Guided Superior Hypogastric Plexus Block

**Figure 2:**
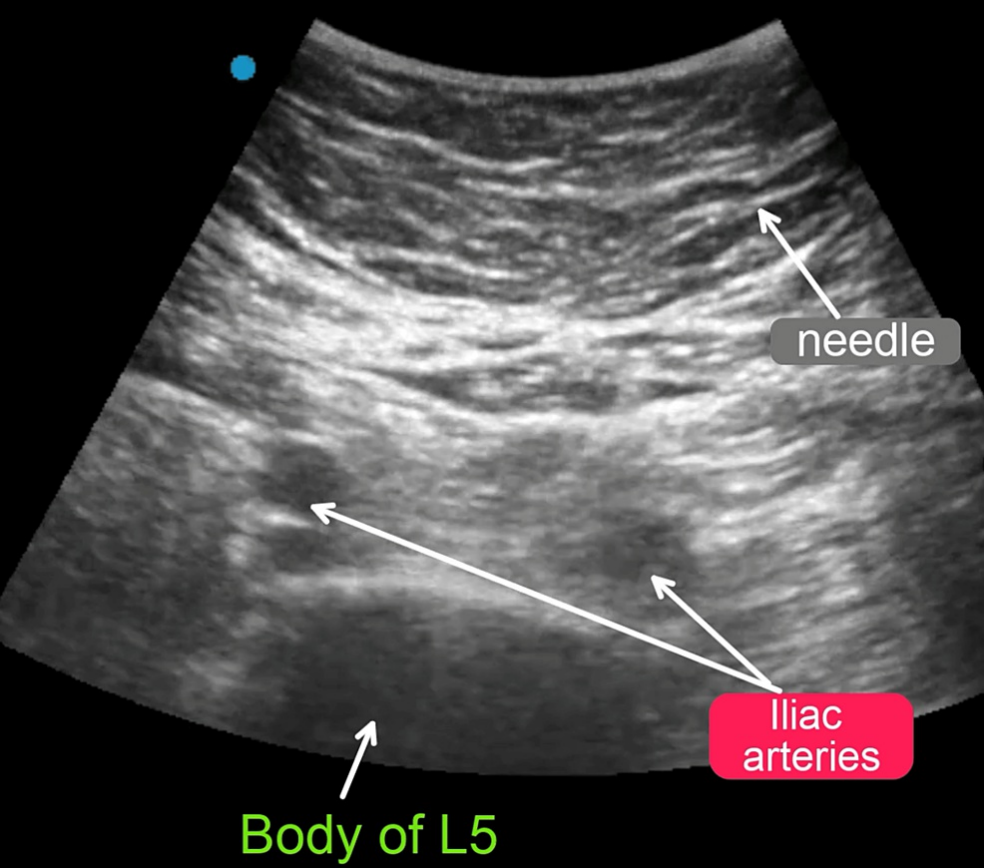
Ultrasonography displaying the location of the superior hypogastric plexus block

The patient was brought to the procedure room, placed in a supine position, and sedated with intravenous propofol. The area was prepped with 2% chlorhexidine gluconate and 70% isopropyl alcohol and draped in a sterile fashion. Injection of 3 ml of 1% lidocaine was used to anesthetize the skin. The body of the L5 vertebrae was identified with a 5-2 MHz curvilinear ultrasound probe, which was placed in the area between the umbilicus and pubic symphysis (Figure 2).

As the iliac arteries divided, over the L5 vertebrae, a unilateral superior hypogastric plexus block was performed. A 22-gauge needle was inserted under direct ultrasonic guidance to get the body of the vertebrae. After negative aspiration of blood, a solution of 15 ml of 0.5% bupivacaine and 40 mg of methylprednisolone was incrementally injected, with intervening aspiration being negative for blood (Video 2).

**Video 2:** Ultrasound-guided superior hypogastric plexus block Permission for use granted by the American Society for Post Surgical Pain

Case 1

The first case is a 28-year-old female who presented to the clinic with a three-year history of IC/BPS and vaginal introitus pain. At the time of presentation, she reported urinary frequency several times per hour and a constant, burning pain involving the vagina and perineum with a severity of 7/10 on the numerical rating scale (NRS). Extensive workup for urinary tract infection over this period was consistently negative. She was previously started on amitriptyline and hydroxyzine for the management of IC/BPS, without significant pain relief. Two weeks prior to the clinical presentation, she started pelvic floor physical therapy (PT) and reported only a minimal improvement in symptoms. In the setting of this patient’s IC/BPS being refractory to conservative therapies, she was scheduled for bilateral pudendal nerve blocks with local anesthetic and steroid, as detailed above. Two weeks following the procedure, the patient followed up in the clinic and reported 30-50% sustained pain relief, along with a decreased urinary frequency of once per hour. The patient was advised to continue pelvic floor physical therapy and keep a pain diary of daily symptoms. Seven weeks following the procedure, the patient continued to report sustained pain relief with improved control of urinary symptoms. She will continue to monitor symptoms and return later, if needed, for repeat bilateral pudendal nerve blocks and/or a superior hypogastric plexus block.

Case 2

The next case is a 59-year-old female with a history of IC/BPS, vulvodynia, and lichen planus who presented to the clinic for chronic pelvic pain and urinary frequency. Seven years prior, the patient reported a gradual onset of dysuria and bladder pain and sought medical evaluation. Urinalysis was negative for infection, and cystoscopy confirmed the diagnosis of IC/BPS. The patient had been evaluated by numerous physicians, exhausted a variety of pharmacologic agents, and participated in online support groups without relief. During this roughly six-year period, symptoms were severe but steady, until she experienced an exacerbation 10 months prior to visiting the clinic. The patient described a more severe burning pain in her pelvic region, 9/10 in severity on the NRS, and worsening of urinary symptoms, voiding more often than once per hour with frequent nocturia. Once very active and walking five miles per day, the patient now reported being unable to walk further than a quarter mile without experiencing excruciating pain. At the time of presentation, the patient was not taking any medications but was undergoing PT with only minimal symptomatic relief. She underwent bilateral pudendal nerve blocks with local anesthetic and steroids using the same procedural techniques detailed above. Three days following the procedure, the patient reported greater than 50% improvement in symptoms. For the first time in several years, she described the absence of intense burning pain while voiding and endorsed a more vigorous urinary stream. The patient was advised to continue PT and keep a pain diary of daily symptoms. One week following the procedure, she reported her pain at or less than a 4/10 in severity on the NRS with intermittent, rather than constant, symptoms.

Case 3

The next case is a 41-year-old male with a history of IC/BPS who presented to the clinic for chronic pelvic pain and urinary frequency and urgency of 12 years duration. At the time of presentation, he was taking antibiotics for possible prostatitis, without symptomatic relief, and had failed trials of amitriptyline, hydroxyzine, and other pharmacologic agents. He had recently started PT and reported two days of moderate but short-lived relief after each session. The pain was described as an uncomfortable, pressure-like sensation in the perineum with a severity of 6/10 on the NRS. He reported voiding more than once per hour with incomplete bladder emptying and frequent nocturia. The patient was deemed a candidate for bilateral pudendal nerve blocks with local anesthetic and steroids in the setting of failed conservative treatment measures. He initially underwent two diagnostic bilateral pudendal nerve blocks, spaced one week apart, that provided five months of moderate to significant pain relief. At the five-month interval, he underwent two additional bilateral pudendal nerve block procedures in the span of two weeks, which provided significant pain relief for an additional four months. Another bilateral pudendal nerve block was then performed, and the patient reported greater than 50% sustained pain relief for nearly one year. Since the patient still had residual pain after pudendal nerve blocks, when he returned to the clinic at that time, a decision was made to proceed with a superior hypogastric plexus block. After the procedure, the patient reported greater than 80% sustained pain relief, reduced bladder spasms, and decreased urinary frequency for a duration of five months. When he returned to the clinic, he underwent an additional bilateral pudendal nerve block and a second superior hypogastric plexus block three weeks later. Three months following the superior hypogastric plexus block, the patient reported a drastic reduction in symptoms with pain of 4/10 or less on the NRS and voiding less than once per hour. He stated that the peripheral nerve block procedures typically provide relief for six-eight months, and he will follow up in the clinic at that time to determine the next steps in management.

## Discussion

Despite multiple modalities available to manage IC/BPS, no optimal therapy currently exists. The choice of therapy is guided by patient-specific factors, as well as a consideration of appropriate risks and benefits. Behavioral and non-pharmacologic therapies are the safest and least invasive interventions, but their efficacy is not well supported by the literature. A review of oral medications showed varying levels of success [8,9], but the presence of potential adverse effects may limit their use. A recent meta-analysis on the efficacy of intravesical therapy deemed hyaluronic acid superior to other treatment options, though the authors report modest symptomatic improvement among all interventions, including placebo groups [10]. In refractory cases, more invasive procedures include hydrodistension, neuromodulation, and diversion with or without cystectomy, but these options may lead to complications and poor postoperative outcomes.

In the cases presented here, the patients demonstrated little relief with prior conservative measures including behavioral and dietary modifications, pelvic floor physical therapy, and oral medications. Subsequently, we performed bilateral pudendal nerve blocks and/or ultrasound-guided superior hypogastric plexus blocks, which the current guidelines do not address, rather than advancing to more invasive procedures.

A description of the rationale for each procedural technique is provided here. Pudendal nerve blocks via fluoroscopic- [11], ultrasound- [12], CT- [13], and MRI-guided [14] approaches have been described with varying levels of success. Ultrasound-guided transperineal pudendal nerve blocks in children [15] seem relatively easy and effective, but in adults, this becomes challenging due to poor tissue penetration with low-frequency ultrasound and the presence of scarring. In addition, most pain physicians are not familiar with the transrectal and transvaginal approaches, which suit gynecological, urological, and colorectal surgeons. Hence, there is a need for a simple and safe alternative approach to pudendal nerve blocks. Of note, an ischiorectal surgical approach for pudendal nerve schwannoma resection was described as a safer approach, since there is a paucity of vessels and an abundance of fat in the ischiorectal fossa [16].

Ultrasound-guided superior hypogastric plexus blocks offer the following advantages over fluoroscopy: zero exposure to radiation, a supine posture that provides easy access to the airway for the anesthetist, single-needle procedure, minimal risk of vascular puncture, and shorter procedure duration. The anterior approach, like the fluoroscopic-guided posterior approach, carries the risk of vascular puncture and bowel and bladder perforation. However, human studies have not reported any incidence of such injury [17], and the authors have not encountered any complications either.

Bladder control and micturition are complex processes mediated by input from the brain, spinal cord, and peripheral nerves. The hypogastric nerves, which originate from the superior hypogastric plexus, contain sympathetic fibers that innervate the base of the bladder and urethra. Activation of these nerves results in bladder smooth muscle relaxation and urethral smooth muscle contraction, effectively storing urine. The external urethral sphincter, responsible for voluntary urinary control, receives somatic motor input from the pudendal nerves. When these somatic nerves are stimulated, the result is the contraction of the external urethral sphincter and the release of urine. Together, the hypogastric and pudendal nerves maintain tight regulatory control of micturition.

The pudendal nerves carry motor and sensory axons originating from the sacral spinal nerves S2-S4. These bilateral nerves form terminal branches, which innervate regions of the perineum, external genitalia, anus, and external urethral and external anal sphincters. Due to the mixed motor and sensory components, damage to or overactivity of these nerves can lead to visceral pelvic pain and urinary dysfunction. In this context, peripheral nerve blocks of the pudendal nerves may be an ideal target to reduce pelvic pain [6,18]. A retrospective study of 84 patients with IC/BPS demonstrated significant improvements in pain and function after six weeks of weekly pudendal and posterior femoral cutaneous nerve blocks, in combination with pelvic floor trigger point injections and physical therapy [19]. However, analysis of pain and function was only conducted at the three-month consult; the efficacy of the treatment protocol was not assessed past this time point. In addition, weekly nerve block injections may be inconvenient or even unfeasible for some patients, limiting the utility of these results.

The superior hypogastric plexus is located around the L5-S1 intervertebral disc space anterior to the bifurcation of the aorta. The superior hypogastric plexus then bifurcates into left and right hypogastric nerves, which transmit predominantly sympathetic fibers to the pelvis. Blockade of the plexus may serve a therapeutic function to reset the neurogenic input to the bladder, helping to alleviate pelvic pain and symptoms of urinary urgency, frequency, or nocturia associated with IC/BPS. In a female patient with IC/BPS (Kim et al.), diagnostic superior hypogastric plexus block followed by two sessions of pulsed radiofrequency treatment of the superior hypogastric plexus provided symptomatic relief for over two years [20].

However, due to limited data supporting the use of pudendal nerve blocks or superior hypogastric plexus blocks for the management of IC/BPS, their indication has not yet been formally established. Here, we report improvements in pain, urinary tract symptoms, and functionality following pudendal nerve blocks and superior hypogastric plexus blocks in three patients with IC/BPS refractory to conservative therapy. These nerve blocks are in the best interest of both patients and insurance providers, as they allow patients to avoid surgical interventions and long-term medication management. Our findings provide promising support for the use of these therapeutic interventions in patients with IC/BPS, though prospective randomized controlled studies are necessary to fully evaluate the clinical safety and efficacy of these procedures.

## Conclusions

Bilateral pudendal nerve blocks and ultrasound-guided superior hypogastric plexus blocks appear to be safe and efficacious in reducing pain and urinary symptoms in patients with refractory IC/BPS. With the updated AUA guidelines that recommend the use of multimodal pain management options, as opposed to the prior tier-based stepwise approach, these peripheral nerve block procedures should be considered earlier in the management process. These interventions are quick, safe, and easy to perform and provide patients with both short- and long-term relief of unremitting symptoms.
